# Lignin Structural Characterization and Its Antioxidant Potential: A Comparative Evaluation by EPR, UV-Vis Spectroscopy, and DPPH Assays

**DOI:** 10.3390/ijms25169044

**Published:** 2024-08-21

**Authors:** Tina Ročnik Kozmelj, Maxim A. Voinov, Miha Grilc, Alex I. Smirnov, Edita Jasiukaitytė-Grojzdek, Lucian Lucia, Blaž Likozar

**Affiliations:** 1 Department of Catalysis and Chemical Reaction Engineering, National Institute of Chemistry, Hajdrihova 19, 1001 Ljubljana, Slovenia; 2 Department of Chemistry, North Carolina State University, 2620 Yarbrough Drive, Raleigh, NC 27695-8204, USA; mvoynov@ncsu.edu (M.A.V.); aismirno@ncsu.edu (A.I.S.); lalucia@ncsu.edu (L.L.); 3 Department of Forest Biomaterials, North Carolina State University, 2820 Faucette Drive, Raleigh, NC 27695-8005, USA

**Keywords:** organosolv pulping, lignin fraction, lignin oligomers, antioxidant activity, EPR, DPPH assay

## Abstract

The natural aromatic polymer lignin and its lignin-like oligomeric fragments have attracted attention for their antioxidant capacity and free radical scavenging activities. In this study, a 2,2-diphenyl-1-picrylhydrazyl (DPPH) assay was employed to assess the antioxidant capacity of fractionated and partially depolymerized organosolv lignin by electron paramagnetic resonance (EPR) and UV-Vis spectroscopy. The results show significant antioxidant activity for both the lignin and oligomeric fragments, with the EPR measurements demonstrating their efficiency in quenching the free radicals. The EPR data were analyzed to derive the kinetic rate constants. The radical scavenging activity (RSA) of lignins was then determined by UV-Vis spectroscopy and the results were compared with the EPR method. This two-method approach improves the reliability and understanding of the antioxidant potential of lignin and its derivatives and provides valuable insights for their potential applications in various industries, including pharmaceuticals, food preservation, and cosmetics.

## 1. Introduction

Lignin, a natural polymer abundant in plant cell walls, is a promising antioxidant with many potential applications, including biomedicine and cosmetics. The antioxidant properties of lignins are attributed to its unique chemical structure whose abundance of phenolic hydroxyl groups provide for the efficient scavenging of free radicals. The radical scavenging activity is even more pronounced for lignin oligomers, which were proposed for use in cosmetic formulations as an environmentally friendly alternative to synthetic antioxidants [1,2,3,4,5].

The evaluation of compounds for antioxidant activity is a complex task for which a number of analytical methods and assays have been developed over the years (e.g., 2,2-diphenyl-1-picrylhydrazyl (DPPH), 2,2′-azino-bis(3-ethylbenzothiazoline-6-sulfonic acid) (ABTS), ferric ion-reducing antioxidant potential (FRAP), and Folin–Ciocalteu (FC) assays). Typically, antioxidant properties are assessed by analyzing competitive reactions between reactive oxygen species and an antioxidant or a probe, where the oxidation state of the latter is monitored spectroscopically. Currently, there is no consensus on the best single method to characterize compounds for the radical inhibition reactions [6,7,8]. For these reasons, several researchers began employing and correlating the results of different methods [6,7]. Among the techniques currently in use to monitor and compare antioxidant activity and detect free radicals in bio-based materials, electron paramagnetic resonance (EPR) spectroscopy has emerged as one of the most efficient and informative techniques [9]. The EPR method provides direct measurements of concentrations of free radicals, and an analysis of the EPR spectra yields magnetic parameters (e.g., *g*-factor and, if observed, hyperfine coupling constants of the electronic spins with the nuclear spins nearby), which can then be used to identify the chemical nature of the observed paramagnetic species [10].

Until now, the antioxidant activity of lignin has been evaluated by a number of methods. However, a statistical analysis revealed that the DPPH assay (R^2^ < 0.55) does not correlate with other assays using UV-Vis spectroscopy methods, such as ABTS, FRAP, and Folin–Ciocalteu assays [7,8]. On the other hand, a positive correlation between the DPPH and ABTS assays was demonstrated using the EPR measurement (R^2^ = 0.88) [8]. Therefore, this study emphasizes a possibility for correlating the radical scavenging activity of lignin using a DPPH radical solution and two analytical techniques—EPR and UV-Vis spectroscopy.

The present study aims to investigate, compare, and correlate the radical scavenging activity of different hardwood organosolv lignins and lignin-like oligomeric samples with different structural characteristics. Through a comprehensive investigation of the correlations and the effects of specific residues in the lignin structure, we aimed to tune the antioxidant capacity of lignins. In contrast to previous studies that lacked the comparison of multiple methods to evaluate the radical scavenging activities of the samples, we present an approach that combines two analytical techniques, EPR spectroscopy and UV-Vis spectroscopy, and emphasize the importance of using EPR as a complementary technique to confirm the results from UV-Vis measurements. Such a combined approach has a special significance because the reliability of a UV-Vis-based DPPH assay was recently questioned [11,12,13,14,15,16].

## 2. Results and Discussion

### 2.1. Variations in Lignin Molecular Weight and Functionality

**Figure 1:**
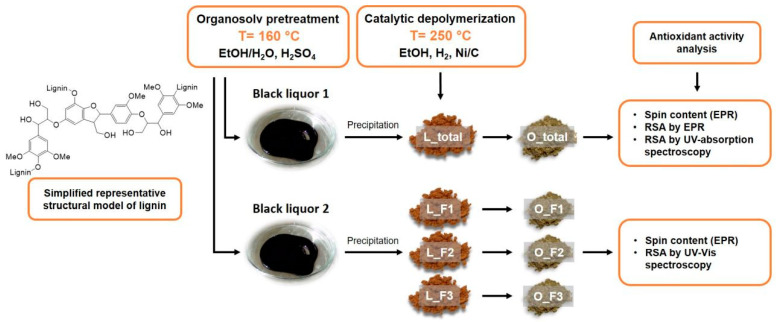
Overview of lignin and oligomer samples tested for antioxidant activity.

The lignin fractions assigned as L_F1, L_F2, and L_F3 were obtained by fractional lignin precipitation with a stepwise modification of the solubility parameters by adding water as an antisolvent [17]. Organosolv lignin (L_total) was isolated as a reference material to evaluate the effect of the fractionation protocol on the variations in the lignin molecular weight and functionality (Figure 1). The molecular weight of the lignin fractions determined by SEC and the content of the aliphatic and phenolic OH groups (lignin functionality) measured by ^31^P-NMR are listed in Table 1. The lignin fractions analyzed in this study had an average molecular weight (M_w_) from 6950 Da (L_F1) to 1850 Da (L_F3), with the highest molecular weight fraction having a higher dispersity of 2.9 and a lower phenolic OH group content of 1.99 mmol g^−1^. Among the lignin fractions, L_F3 had the highest content of phenolic and aliphatic OH groups of 2.70 mmol g^−1^ and 1.84 mmol g^−1^, respectively. Although all the fractions had comparable content of aliphatic OH groups, the change in the content of phenolic OH groups led to a significant decrease in the ratio between the aliphatic and phenolic OH groups with a reduced M_w_ and dispersity of the lignin fractions. The produced lignin as the reference material (L_total) was found to have an M_w_ close to that of L_F2 with an M_w_ of 3400 Da and phenolic OH group content of 2.28 mmol g^−1^ but with a higher dispersity of 2.3 and a slightly higher content of aliphatic OH groups of 1.94 mmol g^−1^ (Table 1). Variations in the molecular weight and the content of the aliphatic and phenolic OH groups were also observed in the oligomeric fragments (O_F1, O_F2, and O_F3) obtained after partial lignin depolymerization. Partial lignin depolymerization afforded oligomeric fragments with pronounced changes in the M_w_, dispersity, and content of the aliphatic and phenolic OH groups. Overall, the M_w_ decreased from 3400 Da to 2000 Da (L_total), the dispersity of the lignin decreased from 2.3 to 1.7, and the content of the aliphatic OH groups reduced from 1.94 mmol g^−1^ to 0.67 mmol g^−1^, while the oligomeric fragments showed a higher content of phenolic OH groups. The change in the ratio between the aliphatic and phenolic OH groups is the result of the successful cleavage of the β-ether bond within the lignin macromolecule. Similarly, the change in the functionality of the lignin after partial lignin depolymerization was described in detail for organosolv acetone lignin by Smit et al. [18]. Figure 1 provides an overview of the lignin, lignin fractions, and oligomeric fragments used in this study to test their antioxidant capacity by EPR and UV-Vis spectroscopy.

### 2.2. Measurement of Spin Content in Lignins by EPR

Spin content refers to the number of unpaired electrons in the material structure that indicates the presence of reactive phenolic hydroxyl groups usually associated with the antioxidant capacity of the material [6,19,20]. It was reported that the spin content in lignins is attributed to the presence of specific functional groups in the structure and quinone-type and phenolic residues in particular. Free radical centers in lignin could also be formed in the course of radical reactions occurring during the fractionation of lignocellulosic biomass or other degradation processes, e.g., depolymerization [9].

**Figure 2:**
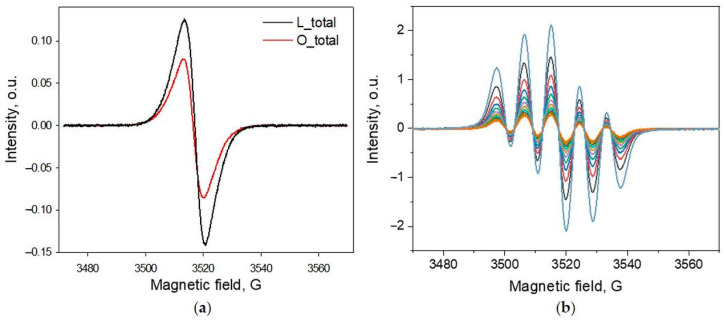
(**a**) The typical X-band EPR spectra of the lignin (L_total) and the corresponding oligomers (O_total) recorded at room temperature (the spectra intensities were normalized by the sample weight); (**b**) a series of sequential X-band EPR spectra of the DPPH dioxane solution in the presence of lignin (L_total) over a period of 40 min (from black to orange lines), while the initial intensity of the DPPH signal was 2.11 (blue line).

EPR spectroscopy provides structural information on the chemical nature of the free radical centers and allows for accurate free radical quantification [9,20]. The X-band (9.5 GHz) CW EPR spectra of the lignin powder samples showed broad (Δ*B*_pp_ = 6.46 ÷ 7.56 G, Figure 2a, Table 2) single-line EPR spectra with no resolved hyperfine structure and *g*-factors ranging from 2.0040 to 2.0043 characteristic of oxygen-centered radicals [21,22,23]. The highest *g* value amongst the lignin fractions was found in fraction L_F3 (*g* = 2.0043). Amongst the oligomeric samples, the highest *g* value was found in fraction O_F3 (*g* = 2.0042). Because L_F3 and O_F3 exhibit the highest content of phenolic OH groups (Table 1), we can assume that the oxygen-centered radicals originating from phenolic residues become predominant in the late fractions. The dominance of phenolic residues in the late fractions manifested itself in major changes in the solvent polarity required to induce precipitation during fractionation; therefore, a higher amount of water was used [24,25]. Interestingly, the spectra peak-to-peak linewidth was found to gradually increase from fraction L_F1 to L_F3 (from 6.46 to 7.56 G) and from oligomeric samples O_F1 to O_F3 (from 6.51 to 6.99 G) (Table 2). Considering the *g*-strain and unresolved hyperfine couplings to be the major factors contributing to the EPR line broadening, we can assume that the structure of the lignin matrix in the late fractions becomes more irregular, which increases the variations in the orientation of the paramagnetic centers. To quantify the spin concentrations in both the lignin and oligomeric samples, double integration of these EPR spectra was employed. The results are summarized in Table 2. Notably, it was observed that the lignin and lignin fractions exhibited a higher spin content compared to the corresponding oligomeric fragments after depolymerization. Lignin (L_total) was found to have a spin concentration of 9.9 × 10^19^ spins g^−1^, while the oligomeric sample (O_total) had a spin content of 6.6 × 10^19^ spins g^−1^. The lignin fractions and the corresponding oligomers also exhibited clear differences in the spin content. Specifically, the lignin fraction F1 (L_F1) and oligomer F1 (O_F1) showed the lowest spin content of 6.8 × 10^19^ spins g^−1^ and 5.7 × 10^19^ spins g^−1^, respectively, whereas the lignin fractions F2 (L_F2) and F3 (L_F3) and/or oligomeric samples (O_F2 and O_F3) exhibited an increased spin content, e.g., 13.4 × 10^19^ spins g^−1^ for L_F3 and 11.4 × 10^19^ spins g^−1^ for the O_F3 samples. The results of the ^31^P-NMR measurements show that the late lignin and oligomer fractions (L_F2, L_F3, O_F2, and O_F3) had a noticeably higher content of phenolic OH groups (Table 1), compared to the L_F1 and O_F1 fractions. That is, fractionation causes an enrichment of the latter, more soluble fractions in aqueous ethanol, with phenolic components; the presence of the latter ones most likely dictates the solubility of said fractions. This also explains a higher spin count in the late fraction. In support of this speculation, Matos et al. [26] reported that acetone-water fractionalization of lignin produced the most soluble fraction, which was enriched with phenolic components and, as a result, showed higher antioxidant activity. A similar observation was made by An et al. [27], who have found that the fraction that was most soluble in dichloromethane and was obtained upon the fractionation of lignin had the highest phenolic content and the highest antioxidant activity.

**Figure 3:**
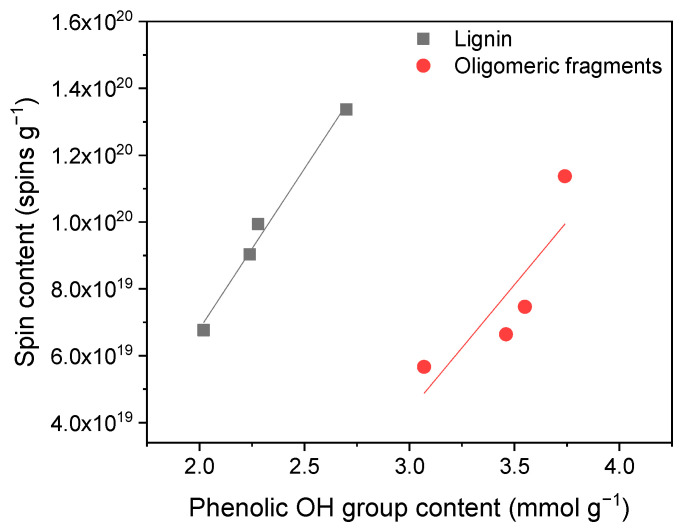
Spin content shows a linear correlation with phenolic OH group content of lignins and oligomers.

Further, we attempted to find out whether there is any relationship between the spin concentration, the molecular weight, and the hydroxyl group content. The results presented in Figure 3 demonstrate that the spin content positively correlates with the content of the phenolic OH groups in the lignin samples and oligomeric fragments. A linear correlation between the spin content and the phenolic OH group content (Figure 2) was found with R^2^ values of 0.99 and 0.80 for the lignin and oligomers, respectively. Moreover, the correlation between the M_w_ of the lignins and oligomeric fragments showed that the lower M_w_ of the samples led to a higher spin content. Thus, the presence of phenolic residues in the chemical structure of lignin samples impacts the spin concentration, which in turn can affect the antioxidant capacity.

### 2.3. Radical Scavenging Activity by EPR

**Figure 4:**
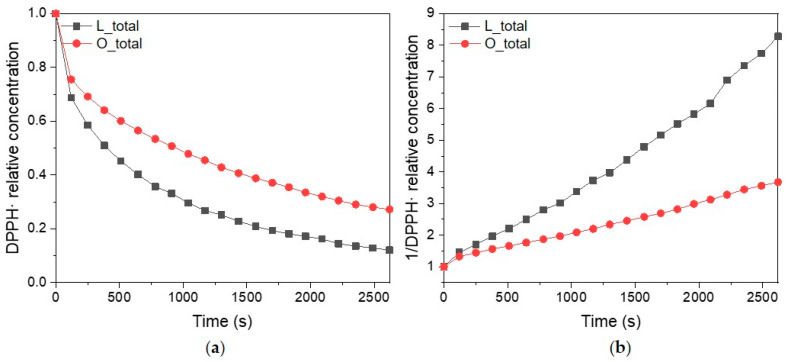
(**a**) The kinetics of DPPH· inhibition by lignin and oligomers; (**b**) the dependence of the reciprocal DPPH concentration on the reaction time.

The presence of unpaired electrons in lignin indicates that it has the potential to neutralize reactive oxygen species and other free radicals. As a polymer possessing multiple phenolic moieties, lignin exhibits antioxidant activity by transferring hydrogen atoms and electrons to free radicals [7,28]. One of the methods that allows for the determination of kinetic parameters of reactive radical inhibition by lignin is CW EPR spectroscopy. Typically, a free radical DPPH is used as a model radical in antioxidant activity assays. The reaction rates of the DPPH radical inhibition by the lignin and the corresponding oligomers were determined by analyzing the intensities of sequentially recorded EPR spectra (Figure 2b). The DPPH decay was found to proceed according to a second-order process. The rate constants k calculated from the plots for the lignin and oligomers were determined to be 0.56 L mol^−1^ s^−1^ and 0.21 L mol^−1^ s^−1^ (Figure 4), respectively. These rate constants indicate a stronger antioxidant capacity of the lignin, which inhibits the DPPH radical two times faster than the oligomers do. Remarkably, the obtained rate constants for radical inhibition by lignin and oligomers correlate with the spin content of the samples measured by an X-band EPR. For lignin, a higher inhibition rate correlated with a higher measured spin content.

### 2.4. Radical Scavenging Activity by UV-Vis Spectroscopy

Unlike the EPR DPPH assay, the UV-Vis-based DPPH assay is more widely used by the scientific community despite the recent attempts to critically re-evaluate this method [13,14,15,16]. Thus, the free radical scavenging activity of lignin and oligomers was also evaluated by following the DPPH decay with UV-Vis spectroscopy. The RSA value reflects the rate of DPPH inhibition and is an indicator of the ability of an antioxidant material to scavenge free radicals. This is evidenced by the decrease in the absorbance of the DPPH solution, which is quantitatively expressed as the RSA value [29,30]. It was found that the characteristics in the lignin fractions affected their RSA (radical inhibition from 29.5 to 41.0%). The variations in the structural characteristics also affected the free radical inhibiting ability of the oligomeric samples; specifically, the radical inhibition was found to be higher (62.9–66.7%) compared to the lignin samples when measured by UV-Vis spectroscopy (Table 2). Therefore, the molecular weight distributions and the content of the phenolic OH groups have a considerable effect on the RSA of the samples. For L_F1, L_F2, and L_F3, a decrease in the M_w_ and an increase in the content of the phenolic OH groups resulted in a pronounced RSA. In contrast, a negligible change in the M_w_ was observed for O_F1, O_F2, and O_F3, while the content of the phenolic OH groups increased, suggesting that phenolic OH groups may contribute more to radical inhibition than the M_w_. However, the lower M_w_ and dispersity (Đ) of oligomers also appear to contribute to better performance, as reflected in a better RSA compared to the lignins. This suggests that lignins with a higher molecular weight have a less uniform and more dispersed structure and, therefore, have a significant number of phenolic OH groups [31,32] that are sterically hindered and inaccessible to DPPH [7]. Thus, the structural differences between lignin and oligomers, such as the phenolic OH group content and their accessibility to radicals, as well as the more tightly packed structure of the high molecular weight lignins, have a major effect on the RSA values. This conclusion is in line with our assumption about a more irregular structure of the lignin–polymer matrix in the late fractions (see above). A similar observation was made by Ponomarenko et al. [33] who were using lignins from different biomass sources and different isolation procedures.

The performance of the DPPH assay was also evaluated using Trolox as a standard (Table 2). The Trolox equivalent antioxidant capacity (TEAC) assay provides an independent assessment of the antioxidant capacity of a material. The lignin and lignin fractions had TEAC values of approximately 0.60 μmol TE mg^−1^, while the oligomeric fragments showed TEAC values of about 1.12 μmol TE mg^−1^, indicating that the lignins (L_total, L_F1, L_F2, and L_F3) examined in this study have a higher antioxidant capacity compared to other previously tested lignins. Previous publications have reported that the antioxidant capacity of different lignins ranged from 0.25 to 0.60 μmol TE mg^−1^ lignins in TEAC values [7,34,35,36]. For example, milled wood lignin and acetic acid lignin from bamboo shoot shell showed antioxidant capacities of 0.27 μmol TE mg^−1^ lignin and 0.63 μmol TE mg^−1^ lignin, respectively [35], while lignosulfonates and lignins isolated by an oxidative alkaline method exhibited an even lower antioxidant capacity of about 0.30 μmol TE mg^−1^ lignin [36]. On the other hand, alkali and alkaline ethanol lignins from bamboo chips showed an antioxidant capacity in the range of 0.28 μmol TE mg^−1^ lignin to 0.51 μmol TE mg^−1^ lignin [34]. The variations in the obtained TEAC values were therefore caused by the isolation and fractionation procedures and the origin of the biomass and thus by differences in the lignin structural characteristics [34,35,36].

### 2.5. EPR vs. UV-Vis Spectroscopy

The DPPH assay for the RSA measurements and antioxidant capacity by EPR and UV-Vis spectroscopy revealed different antioxidant capacities for the lignin and oligomeric fragments. In particular, EPR showed that lignins (L_total, L_F1, L_F2, and L_F3) inhibited the free radicals faster and exhibited higher antioxidant capacity, while UV-Vis spectroscopy, in contrast, showed the oligomeric fragments (O_total, O_F1, O_F2, and O_F3) to be the more effective antioxidant material. Therefore, a further explanation and discussion of the methods used and their reliability for determining the antioxidant capacity of materials is provided. Sanna et al. [11] highlighted different aspects of EPR and UV-Vis spectroscopies and recommended parallel measurements. It was speculated that the colored decomposition products of the studied materials and/or impurities in the DPPH solution may absorb at the same wavelength of 518 nm and interfere with the UV-Vis method. In contrast, none of the DPPH decomposition products are paramagnetic and, therefore, do not contribute to EPR signals. These considerations indicate that the EPR method is more reliable, not affected by the nature of antioxidants, and independent of the chemical composition of the samples [11]. These limitations of the UV-Vis-based DPPH assay and a possibility of obtaining erroneous results require considering the UV-Vis spectroscopic properties of all the components in the sample [12,13]. For this reason, Celiz et al. [12] developed a new methodology to evaluate antioxidant capacity by UV-Vis DPPH assay that is based on using full-range UV-Vis spectra for calculation instead of performing measurements at fixed wavelengths. Such an approach provided reliable results for the determination of the antioxidant capacity of samples with a high absorbance and spectrophotometrically non-active antioxidants. Moreover, it has been reported that both the radical and reduced forms of DPPH contribute to UV-Vis analysis and that the model used for the calculation of DPPH concentration may cause an overestimation of about 7% [14]. On the other hand, the EPR method is independent of the UV-Vis spectroscopic properties of the materials, resulting in fewer discrepancies and values closer to the actual antioxidant capacity of the samples. The direct detection of radicals, the high selectivity, and the ability to identify specific radical types make EPR an excellent method for assessing the antioxidant capacity of samples. These advantages ensure that EPR provides detailed, accurate, and reliable data, which support its performance and increase its value in antioxidant research [37].

Nevertheless, in the parallel measurements, both methods confirmed the antioxidant capacity of the lignin and lignin-derived samples despite the method-related differences and the limitations of the optical detection of DPPH and its decomposition products. The antioxidant capacity of the lignin correlated significantly with the molecular weight and phenolic hydroxyl group content, which is a promising aspect for the isolation, fractionation, and depolymerization of lignin and allows for further manipulations to give a new market value to different lignins. We have proven that lignin fractionation obtained directly from black liquor and the subsequent depolymerization allowed us to manipulate the antioxidant properties of the lignin material for an even more promising and tailored end-use application.

## 3. Materials and Methods

### 3.1. Lignin and Oligomeric Samples Preparation and Characterization

Organosolv lignin was isolated from beech hardwood with 75 vol% aqueous ethanol (1:7 (*w*/*v* ratio), and 1.0% of H_2_SO_4_ was added to catalyze the reaction. The isolation was performed in a 300 mL batch reactor (Parker Autoclave Engineers, Erie, PA, USA) at 160 °C and 1 MPa nitrogen for 1 h. After filtration of the solid particles, the lignin was precipitated by adding water in threefold excess, while the lignin fractions F1, F2, and F3 were precipitated by adding water in three separate quantities of 150 mL. The lignin was collected upon centrifugation, washed with distilled water, and freeze-dried [38].

The oligomeric samples were obtained after the cleavage of β-ether bonds and depolymerization of the isolated lignin samples in a 75 mL batch reactor (Series 5000 Multiple Reactor System, Parr Instrument Company, Moline, IL, USA) with a Ni/C catalyst (Riogen, Monmouth Junction, NJ, USA) at 250 °C and 1 MPa initial hydrogen pressure for 4 h, while the oligomeric fragments were obtained after precipitation with triple excess water at the end of the process [39].

Quantitative ^31^P-NMR (nuclear magnetic resonance) measurements were performed for each lignin and oligomeric sample according to the protocol reported by Meng et al. [40]. This method is based on excessive phosphitylation of phenolic and aliphatic hydroxyl groups of lignin. Thus, in a prior analysis, the samples were dissolved in a deuterated chloroform/pyridine mixture (1:1.6) and treated with 2-chloro-4,4,5,5-tetramethyl-1,2,3-dioxaphospholane as a phosphitylation reagent. The averaged values of three consecutive ^31^P-NMR measurements are reported, while the highest standard deviation was 0.02 mmol g^−1^. Size-exclusion chromatography (SEC) analyses were carried out following the protocol reported by Jasiukaitytė-Grojzdek et al. [38]. The averaged values of three consecutive SEC measurements are reported, whereby the averaged standard deviation was 85 g mol^−1^.

### 3.2. EPR Spin Counting and Radical Scavenging Activity

For the spin counting, X-band continuous wave (CW) EPR spectra from the lignin, lignin fractions, and oligomeric samples were recorded using a Varian (Palo Alto, CA, USA) Century Series E-109 spectrometer (9.5 GHz) interfaced to a PC and an ELEXSYS E580 spectrometer (Bruker Biospin, Billerica, MA, USA) operating at 9.7 GHz. The powder samples were placed into a clear gelatine pill capsule (size 5, XPRS Nutra, West Jordan, UT, USA), and the capsule was inserted into a polypropylene straw (190 × 6 mm) open from both ends. The typical data acquisition parameters were as follows: modulation amplitude, 1 G; time constant, 64 ms; incident microwave power, 2 mW; sweep time, 30 s; and scan width, 100 G. The spin concentrations were calculated from double integrals of the first-derivative EPR spectra using solid 4-hydroxy-2,2,6,6-tetramethylpiperidin-1-oxyl (TEMPOL) as a standard.

The electronic *g*-factors were determined according to [41] using MnO as a standard.

The radical scavenging activity was determined by the decay rate of the EPR signal of 2,2-diphenyl-1-picrylhydrazyl radical (DPPH) in the presence of lignin samples. The CW EPR spectra were recorded using the ELEXSYS E580 spectrometer (Bruker Biospin, Billerica, MA, USA) operating at X-band (9.7 GHz). The data acquisition parameters were as follows: modulation amplitude, 1 G; time constant, 20 ms; conversion time, 39 ms; incident microwave power, 2 mW; sweep time, 40 s; and scan width, 100 G. Typically, 20 consecutive spectra were collected with a 100 ms delay between the spectra; each spectrum was averaged over 3 scans. The measurements were performed in dioxane solution. In a typical EPR experiment, 90 µL of DPPH solution from 1 mM stock in dioxane was added to 10 µL of dioxane solution containing 0.04 mg ml^−1^ of a lignin sample, and the resulting solution was shaken thoroughly. The stopwatch was started immediately. The blank measurement was performed with DPPH solution in dioxane (90 μL) with the addition of pure dioxane (10 μL) and the DPPH intensity value of the blank was 2.11. For the EPR measurements, dioxane solutions were drawn into a 100 µL glass capillary (OD (in) 0.0565, ID (in) 0.0413; Drummond Scientific Company, Broomall, PA, USA). All the EPR measurements were performed at 21 °C. The peak-to-peak intensities of the central EPR spectral component were measured and plotted against the reaction time, taking into account the time delay [42].

### 3.3. Radical Scavenging Activity by UV-Vis Spectroscopy

The antioxidant capacity by UV-Vis spectroscopy with a DPPH assay was conducted as described by Alzagameem et al. [43] and Rumpf et al. [7]. Briefly, 0.02 mL of 1 mg ml^−1^ lignin solution in dioxane was mixed with 0.78 mL DPPH radical solution (6 × 10^−5^ M in dioxane), and the absorption at 518 nm was measured using a BioTek microplate reader (Agilent, Santa Clara, CA, USA) after 30 min incubation. The radical scavenging activity (RSA) was calculated using Equation (1), where A_sample_ is the absorbance of the tested samples and A_blank_ is the absorbance of the DPPH solution with pure dioxane added instead of the lignin solution. Three parallel measurements were performed for each lignin and oligomeric sample and the averages are reported in this work. The maximum standard deviation of the RSA values was 4.6%. Trolox standards in the range of 25–250 mg L^−1^ were used for the calibration curve from which the concentration of the Trolox equivalent (TE) was calculated. The Trolox equivalent antioxidant capacity (TEAC) was calculated using Equation (2), where C_TE_ is the concentration of Trolox equivalent, M_Trolox_ = 250.3 g mol^−1^ is the molar mass of Trolox, and C_lignin_ is the concentration of the lignin solution.

RSA (%) = (1 − (A_sample_/A_blank_)) × 100%, (1)

TEAC (μmol mg^−1^) = C_TE_/(C_lignin_ × M_Trolox_). (2)

## 4. Conclusions

In this work, lignin, lignin fractions, and the corresponding oligomeric fragments obtained via catalytic depolymerization were analyzed. The pulping and fractionation processes were found to influence the distribution of the lignin structural characteristics, which were then related to the spin content of the analyzed samples. The investigation of the relationship between the lignin structural features and the spin content revealed a linear correlation with the content of the phenolic OH groups in the lignins and oligomers, with a higher spin content observed in the samples with a lower M_w_. The EPR-based DPPH assay was employed to determine the kinetic parameters of the radical inhibition by the lignins. The inhibition was found to proceed according to the second-order process, with the rate constant for lignin being 0.56 L mol^−1^ s^−1^ over the preformed oligomeric fragments with the rate constant of 0.21 L mol^−1^ s^−1^. However, the calculated RSA from the DPPH assay measured by UV-Vis spectroscopy showed a different trend of the antioxidant capacities of the lignins and oligomeric fragments, with the oligomeric fragments exhibiting a higher RSA compared to the lignins. The antioxidant capacity of L_total in Trolox equivalents was measured to be 0.60 μmol mg^−1^, while for O_total it was 1.12 μmol mg^−1^. The evaluation of the Trolox equivalent antioxidant capacity of the studied lignin samples (L_total, L_F1, L_F2, and L_F3) by UV-Vis spectroscopy showed that they have a higher antioxidant capacity compared to other previously published results on the antioxidant capacity of lignins (e.g., milled wood lignin, lignosulfonates, and alkali and alkaline lignin). Nevertheless, the experimental limitations and errors in the measurement by UV-Vis spectroscopy raise legitimate doubts about the reliability of the TEAC results as well. Given the limitations of the UV-Vis method related to the additional absorption of impurities and its color sensitivity, the EPR technique is more suited to obtain reliable results on the antioxidant activity.

## Figures and Tables

**Table 1:** Molecular weight of lignin and oligomeric fragments determined by SEC and their functionality determined by ^31^P-NMR.

Lignin Sample	SEC Results (g mol^−1^)			^31^P-NMR Results (mmol g^−1^)		
	M_w_	M_n_	Đ ^1^	Aliphatic OH	Phenolic OH	Total OH
L_F1	6950	2400	2.9	1.82	1.99	3.81
L_F2	3450	1800	1.9	1.81	2.24	4.07
L_F3	1850	1150	1.6	1.84	2.70	4.57
L_total	3400	1450	2.3	1.94	2.28	4.25
O_F1	2150	1200	1.8	0.51	3.07	3.58
O_F2	2100	1150	1.8	0.62	3.55	4.28
O_F3	1600	1000	1.6	0.63	3.74	4.39
O_total	2000	1150	1.7	0.67	3.36	4.14

^1^ Dispersity.

**Table 2:** Spin content and radical scavenging activity of lignin and oligomeric samples.

Lignin Sample	EPR Results			DPPH Assay ^2^	
	Spin Content ^1^ (spins g^−1^)	*g*-Factor	Δ*H*_pp_ (G)	RSA (%)	TEAC (μmol mg^−1^)
L_F1	6.8 × 10^19^	2.0040	6.45	29.6	0.47
L_F2	9.0 × 10^19^	2.0042	6.94	34.1	0.55
L_F3	13.4 × 10^19^	2.0043	7.56	41.0	0.67
L_total	9.9 × 10^19^	2.0042	7.10	36.6	0.60
O_F1	5.7 × 10^19^	2.0040	6.51	62.9	1.09
O_F2	7.5 × 10^19^	2.0041	6.88	64.6	1.12
O_F3	11.4 × 10^19^	2.0042	6.99	66.7	1.17
O_total	6.6 × 10^19^	2.0041	6.93	64.6	1.12

^1^ Standard used for calculation: TEMPOL. ^2^ Measured by UV-Vis spectroscopy.

## Data Availability

The raw data supporting the conclusions of this article will be made available by the authors on request.
